# Perturbing BEAMs: EEG adversarial attack to deep learning models for epilepsy diagnosing

**DOI:** 10.1186/s12911-023-02212-5

**Published:** 2023-07-06

**Authors:** Jianfeng Yu, Kai Qiu, Pengju Wang, Caixia Su, Yufeng Fan, Yongfeng Cao

**Affiliations:** School of Big Data and Computer Science, Guizhou Normal University, Guiyang, 550025 China

**Keywords:** EEG, BEAMs, Deep learning model, Epilepsy, Adversarial attack, Sparse attack

## Abstract

Deep learning models have been widely used in electroencephalogram (EEG) analysis and obtained excellent performance. But the adversarial attack and defense for them should be thoroughly studied before putting them into safety-sensitive use. This work exposes an important safety issue in deep-learning-based brain disease diagnostic systems by examining the vulnerability of deep learning models for diagnosing epilepsy with brain electrical activity mappings (BEAMs) to white-box attacks. It proposes two methods, Gradient Perturbations of BEAMs (GPBEAM), and Gradient Perturbations of BEAMs with Differential Evolution (GPBEAM-DE), which generate EEG adversarial samples, for the first time by perturbing BEAMs densely and sparsely respectively, and find that these BEAMs-based adversarial samples can easily mislead deep learning models. The experiments use the EEG data from CHB-MIT dataset and two types of victim models each of which has four different deep neural network (DNN) architectures. It is shown that: (1) these BEAM-based adversarial samples produced by the proposed methods in this paper are aggressive to BEAM-related victim models which use BEAMs as the input to internal DNN architectures, but unaggressive to EEG-related victim models which have raw EEG as the input to internal DNN architectures, with the top success rate of attacking BEAM-related models up to 0.8 while the top success rate of attacking EEG-related models only 0.01; (2) GPBEAM-DE outperforms GPBEAM when they are attacking the same victim model under a same distortion constraint, with the top attack success rate 0.8 for the former and 0.59 for the latter; (3) a simple modification to the GPBEAM/GPBEAM-DE will make it have aggressiveness to both BEAMs-related and EEG-related models (with top attack success rate 0.8 and 0.64), and this capacity enhancement is done without any cost of distortion increment. The goal of this study is not to attack any of EEG medical diagnostic systems, but to raise concerns about the safety of deep learning models and hope to lead to a safer design.

## Introduction

Deep neural network (DNN), have been widely used for the analysis of common signals such as images and speech due to their excellent performance. Ullah et al. proposed a densely attention mechanism-based network (DAM-Net) [1] and a multi-task learning based adversarial semi-supervised framework [2] for COVID-19 detection in chest X-ray. In [3], researchers proposed a novel fully automatic technique for brain tumor regions segmentation by using multiscale residual attention-UNet (MRA-UNet). To help diagnose brain disorders, Hossain et al. [4] and Ding et al. [5] proposed the use of convolutional neural networks (CNN) to extract temporal features from Electroencephalography (EEG) data of epileptic patients to understand the general structure of seizures. In [6], researchers used 1D CNN to detect EEG spectrograms of epileptic patients. Bashivan et al. [7] proposed a new method for learning feature representations from multichannel EEG time series that preserves the structure of EEG data within space, time, and frequency.

However, DNN can be misled when normal samples become adversarial examples due to the addition of perturbations. Deep learning models have significant security concerns: Szegedy et al. [8] find that adding an imperceptible non-random perturbation to a picture has the potential to arbitrarily change the model's predictions; DNN are also vulnerable to adversarial examples in physical world scenarios [9]; normal speech with adversarial perturbations can be transcribed into any phrase the attacker wishes, and the perturbed speech sounds no different from normal speech [10]. The problems of adversarial attack and defense for medical and physiological DNN models have drawn some researchers' attention [11, 12]. Finlayson et al. [12] have demonstrated that medical deep learning systems are subject to adversarial attacks. Zhang et al. [13] find that adversarial attacks could make visual perception spelling errors or BCI-based wheelchairs out of the control of the person's consciousness.

EEG is the most widely used clinical tool to measure electrical signals of the brain for understanding the physiological and psychological activities of human. From raw EEG signals, it is easy and convenient to detect amplitude features such as spikes, but not so easy to learn other kinds of information such as spatial and frequency features. That is why many studies first extract useful empirical features from raw EEG signals and then put them into deep neural network models alone or together with raw EEG [14, 15].

Brain electrical activity mapping (BEAMs) are topographic maps of brain EEG power of specified rhythms (frequency bands), which visually display the distribution of different spectra and power levels by anatomical sites in the form of brain topography. BEAM is the earliest and most developed technique in quantitative EEG studies, serving as an advanced diagnostic tool for the evaluation of brain disease episodes and subsequent treatment. It has been widely and successfully applied in clinical diagnosis and validated accordingly [16], and its most frequent application is in epilepsy research, particularly as a method to localize epileptic foci and determine the type of epileptic syndrome [17, 18]. A clear advantage of BEAM over EEG is the improved diagnostic accuracy due to the high spatial resolution. The major advantage of BEAM for epileptic focus localization over other neuro-functional conventional studies (such as functional magnetic resonance imaging (fMRI) or positron emission tomography (PET) is the high temporal resolution that allows for separating initiation from rapid propagation of epileptic activity [19, 20].

**Fig. 1:**
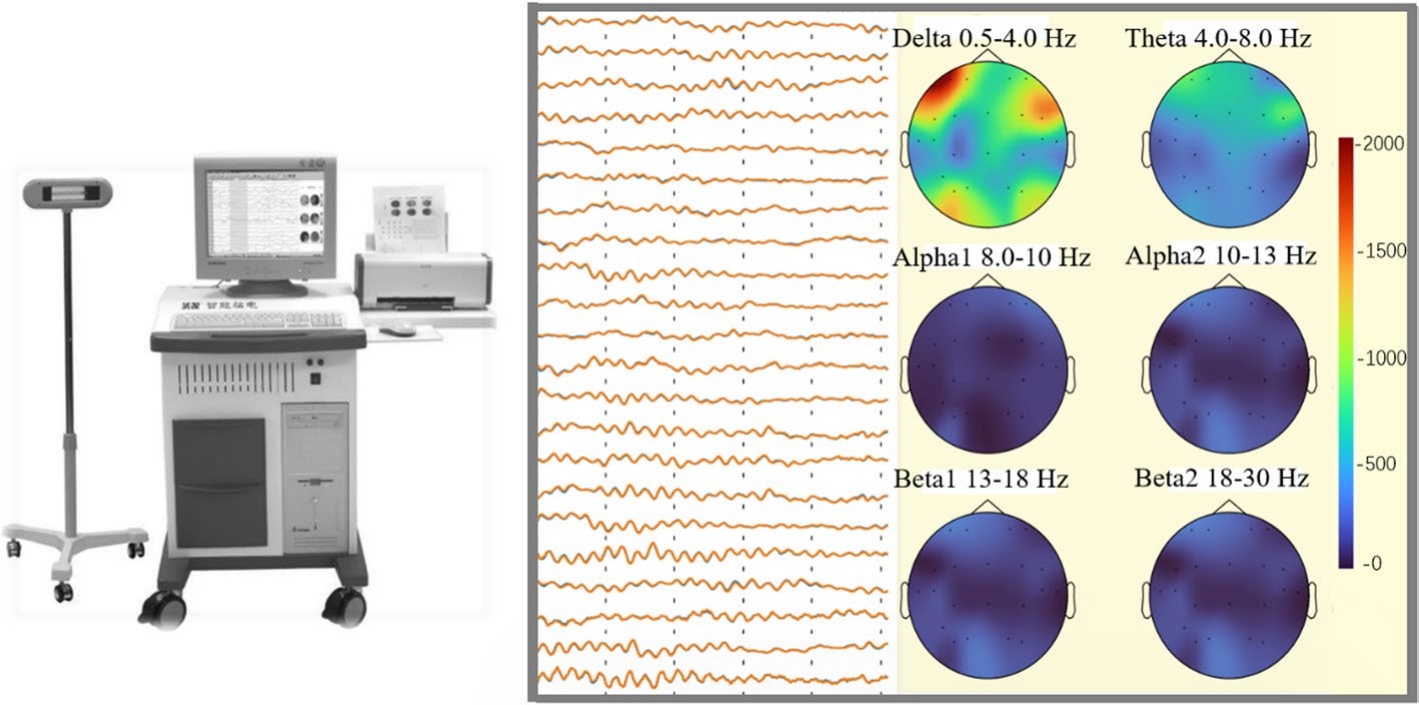
Diagnosis of epilepsy based on EEG (multi-channel waves) and BEAMs (head-shaped color frames)

BEAM has become a very important diagnostic aid in neuroscience. Nevertheless, it was not developed as a replacement for EEG. As shown in Fig. 1, EEG and BEAMs are widely used together by doctors/models to detect the onset of brain diseases, or to analyze brain activities [4, 16]. However, the analysis of adversarial attacks on EEG and BEAM is still very lacking [11, 21, 22], which is far from adequate for the current boom in brain science. Amir et al. [23] first investigated the vulnerability of epilepsy detection systems and showed that adversarial attacks can make epilepsy detection systems to diagnose seizures as non-epileptic. But they only considered SVM-based systems and no studies have yet examined the vulnerability of deep learning models in brain disease (such as epilepsy) diagnosis systems.

In this paper, the vulnerability of deep learning models in the diagnosis system of brain diseases is studied for the first time, and epilepsy diagnosis is used as an example. Currently, studies have been conducted to generate EEG adversarial samples by perturbing the raw EEG signal, EEG frequency and EEG spectrogram. This is the first study that generate EEG adversarial samples by perturbing BEAMs and have done the aggressiveness analysis of these adversarial examples. This paper proposes two methods that generate EEG adversarial samples by perturbing BEAMs, and find that these adversarial attacks can easily lead to misdiagnosis of BEAMs based epilepsy diagnosis. The study exposes an important safety issue in brain disease diagnostic systems and hopefully will lead us to design safer systems.

To summarize, the contributions of this paper are as follows:

1. An EEG white-box dense adversarial attack method are proposed. It generates EEG adversarial samples by imperceptibly perturbing all elements of BEAMs and then converting and adding the perturbation on the BEAMs to raw EEG samples (GPBEAM Section).
2. An EEG white-box sparse adversarial attack method is proposed. It generates EEG adversarial samples by imperceptibly perturbing only partial elements of BEAMs, leaving the attack possibly sparse in the dimension of time slice, rhythm, and electrode (GPBEAM-DE Section).
3. As far as we know, for the first time, EEG adversarial samples are generated by perturbing BEAMs and studied for the adversarial attack analysis of DNN models in brain disease diagnostic systems.
4. The study shows that small perturbations on EEG or BEAMs may lead to misdiagnosis of epilepsy, exposing a critical safety issue in the use of DNN for brain disease diagnosis. The proposed methods can be used to test the vulnerability of existing systems, and to help improve their defense to adversarial attacks.

## Related works

### EEG adversarial attack to DNN architectures

Most of the work on EEG adversarial samples attack models with classical machine learning architecture, such as support vector machine SVM [23], typical correlation analysis [13], and regression [24], but only a few of them attack models with DNN architecture [21, 22], although DNN architecture has been widely studied for EEG signal processing [7].

Jiang et al. [21] and Zhang et al. [22] attack EEG-related models that have raw EEG signals (two-dimensional data of time-channel) as input to internal CNN architectures; Zhang et al. [13] attack frequency-related models that have the frequency (two-dimensional data of frequency-channel) as input to typical correlation analysis. Zhang et al. [22] also attack spectrogram-related models that have the spectrogram (three-dimensional data of time–frequency-channel) as input to internal CNN architecture. In all these works, the perturbation on raw EEG signals could be got by calculating the gradient over the whole pipeline, because all steps are differentiable.

Unlike above works, the work in this paper makes white-box attack to BEAM-related models that have BEAMs (four-dimensional data of time-rhythm-width-height) as input to internal DNN architectures. Because the operation of converting EEG signals to BEAMs is not differentiable, this paper does some special work to convert and add perturbations on the BEAMs to the raw EEG signal. These special works include a sampling operation that select power perturbations for electrodes from the perturbations on BEAMs, an imposing operation that add power perturbations to the frequency domain representation of raw EEG data, and an inversing operation that convert the perturbation-affected frequency domain signals to time domain signals by Inverse Fast Fourier Transform (IFFT) and wavelet packet transform (WPT). Besides CNN architectures, this paper also tests the RNN architecture and a hybrid architecture of CNN and RNN. In addition, a simple modification is proposed to make the method in this paper have aggressiveness to both BEAM-related and EEG-related models, and this capacity enhancement is done without any cost of distortion increment. The most difference between this study and existing studies of EEG white-box adversarial attacks are summarized in Table 1.

**Table 1:** The difference between this study and existing studies of EEG white-box adversarial attacks. In contrast to [13, 23, 24], this paper focuses on the vulnerability of DNN; Compared to [22, 25], in addition to studying the vulnerability of CNNs, this paper also studies the vulnerability of CNN + RNN; Unlike existing studies, this paper generates EEG adversarial samples by perturbing BEAMs, as the input to internal architecture is BEAMs; In addition, this paper examines not only dense attacks, but also sparse attacks

Related studies	Victim model type	The internal architecture of the victim model	Inputs to internal architecture	Type of attack
Zhang et al. [13]	Non-DNN	Canonical correlation analysis; Logistic Regression	EEG, EEG frequency	Dense
Aminifar [23]		SVM	EEG	Dense
Meng et al. [24]		Logistic regression	EEG	Dense
Zhang and Wu [22]	DNN	CNN	EEG, EEG spectrogram	Dense
Feng et al. [25]		CNN	EEG	Sparse
This paper		CNN; RNN + CNN	BEAMs	Dense; Sparse

### EEG sparse adversarial samples

A sparse adversarial sample is a special adversarial sample that requires only a small number of elements perturbed to deceive victim models. With the constrained perturbation size on one element, sparse attacks which perturb a few elements usually have higher stealth and less aggressiveness compared to dense attacks which perturb all elements instead. However, if the information of the features perturbed by a sparse attack is representative of this sample, its aggressiveness could be not much lower than that of dense attacks [26].

The work in this paper is inspired mostly by research in non-EEG fields: Wei et al. [27] argue that in a video classification task, perturbations added to one frame can be passed to the next frames through their time interaction, and therefore, not all frames need perturbation; Su et al. [28] find that attacking single pixels in an image using the Differential Evolution (DE) algorithm [29] can produce adversarial samples; Gao et al. [30] find that in the case of single-pixel attack, if the perturbation overflows, dividing the overflow to adjacent pixels can also produce adversarial samples.

Like Wei et al. [27], this study only perturbs part of the time slices of a sample, resulting in a sparse adversarial sample. Inspired by Su et al. [28], DE is used to select some time slices and electrode channels of BEAMs to generate perturbations. As the number of perturbed elements increases, the efficiency of DE will decrease exponentially. Therefore, this paper only uses DE to perturb partial elements of BEAMs, and let their perturbation overflows to other elements just like the work of Gao et al. [30].

The work of Feng et al. [25] is about EEG sparse adversarial attacks. Through adaptive masking, they automatically select the time step and electrode channel of the perturbation under sparse constraints. Unlike Feng et al., this paper attacks BEAM-related models instead of EEG-related models.

### Extracting EEG rhythms

Extracting basic EEG rhythms, such as Delta (0.5 Hz-4 Hz), Theta (4 Hz-8 Hz), Alpha (8 Hz-13 Hz) and Beta (13 Hz-30 Hz) [31], is the key step to get BEAMs.

There is still disagreement on how to extract rhythms during the conversion of EEG signal to BEAMs. For example [32], use band-pass filtering, [33] use wavelet transforms and [34] use WPT. Wavelet transform is a time–frequency analysis method created to solve the problem of decomposing non-stationary signals and is suitable for feature extraction of non-stationary signals such as EEG due to its multi-resolution characteristics. However, it only subdivides the low-frequency part and not the high-frequency part of the signal, so it does not have a high resolution for the high-frequency part. In contrast, WPT allows the segmented high-frequency part to be subdivided while retaining the advantages of the wavelet transform. Therefore, this paper chooses to use the WPT to extract EEG rhythms in this paper.

## Method

This paper proposes two EEG adversarial sample generation methods: Gradient Perturbations of BEAMs (GPBEAM), and Gradient Perturbations of BEAMs with Differential Evolution (GPBEAM-DE). GPBEAM is a dense attack method. GPBEAM-DE is a sparse attack method that produces perturbations on only a small number of electrode points and assigns perturbations beyond the $\epsilon$ constrain ($\epsilon$ used to ensure that there is little disturbance) to other electrode points with the help of GPBEAM's perturbation symbol information.

### GPBEAM

**Fig. 2:**
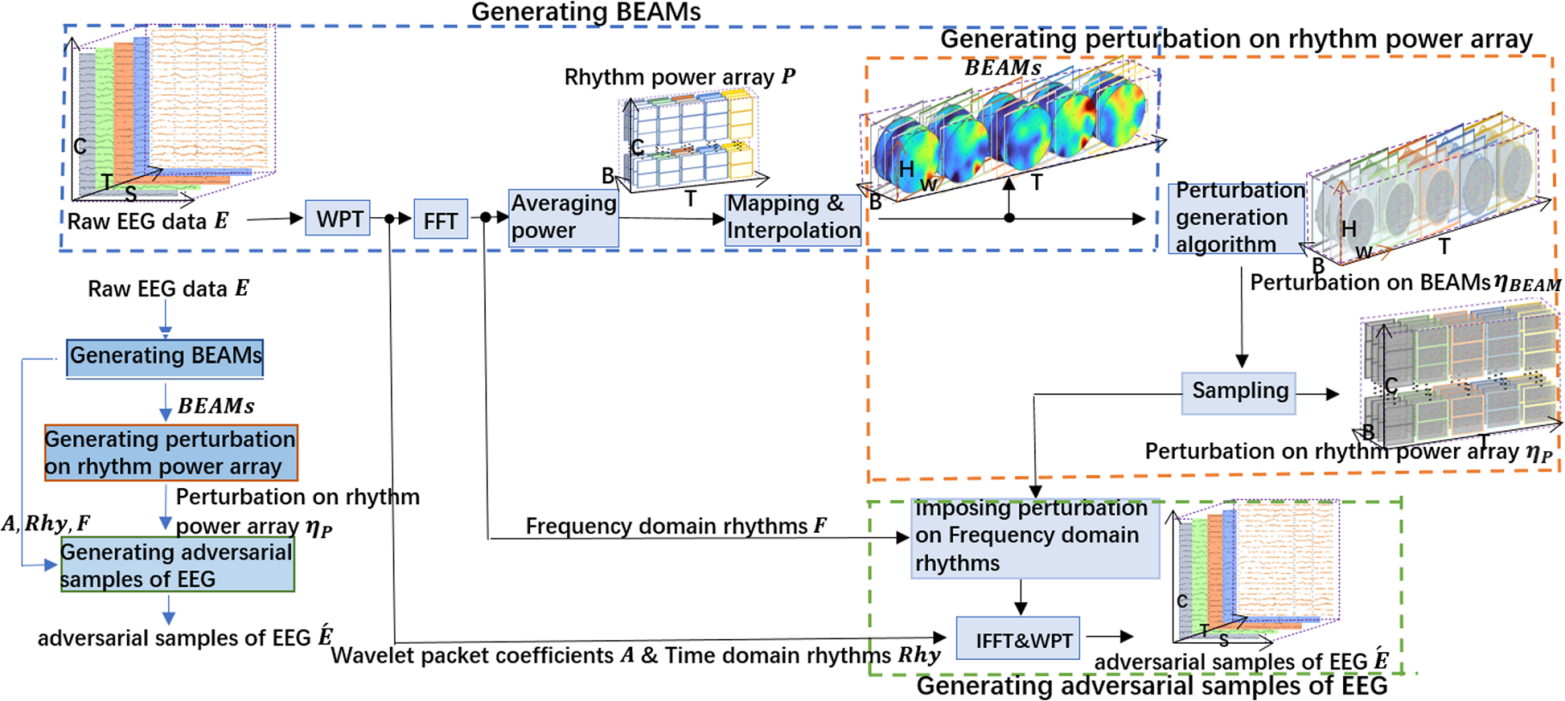
Diagram of GPBEAM. T is the number of time slices; S is the number of voltage values for a single time slice; C is the number of channels, that is, the number of electrodes; B is the number of rhythms extracted; H and W are the length and width of a single BEAM image created from a single time slice of a rhythm

GPBEAM can be divided into three parts (Fig. 2): Generating BEAMs; Generating perturbation on rhythm power array; Generating EEG adversarial samples. In the first part, WPT and FTT are used to extract the spectrum for each of *B* different rhythms from each time slice of the raw EEG data, obtain each rhythm power by averaging the absolute value of the corresponding spectrum, and then construct BEAMs by mapping and interpolating these rhythm power values; In the second part, the adversarial perturbations on BEAMs are first obtained through a perturbation generation algorithm and then reconstructed as the adversarial perturbations on rhythm power array by sampling; In the third part, adversarial perturbations on rhythm power array are added to the frequency domain rhythms, resulting in adversarial samples in the frequency domain. The adversarial samples in the frequency domain are then reconstructed into EEG adversarial samples by IFFT and WPT.

#### Generating BEAMs

**Fig. 3:**
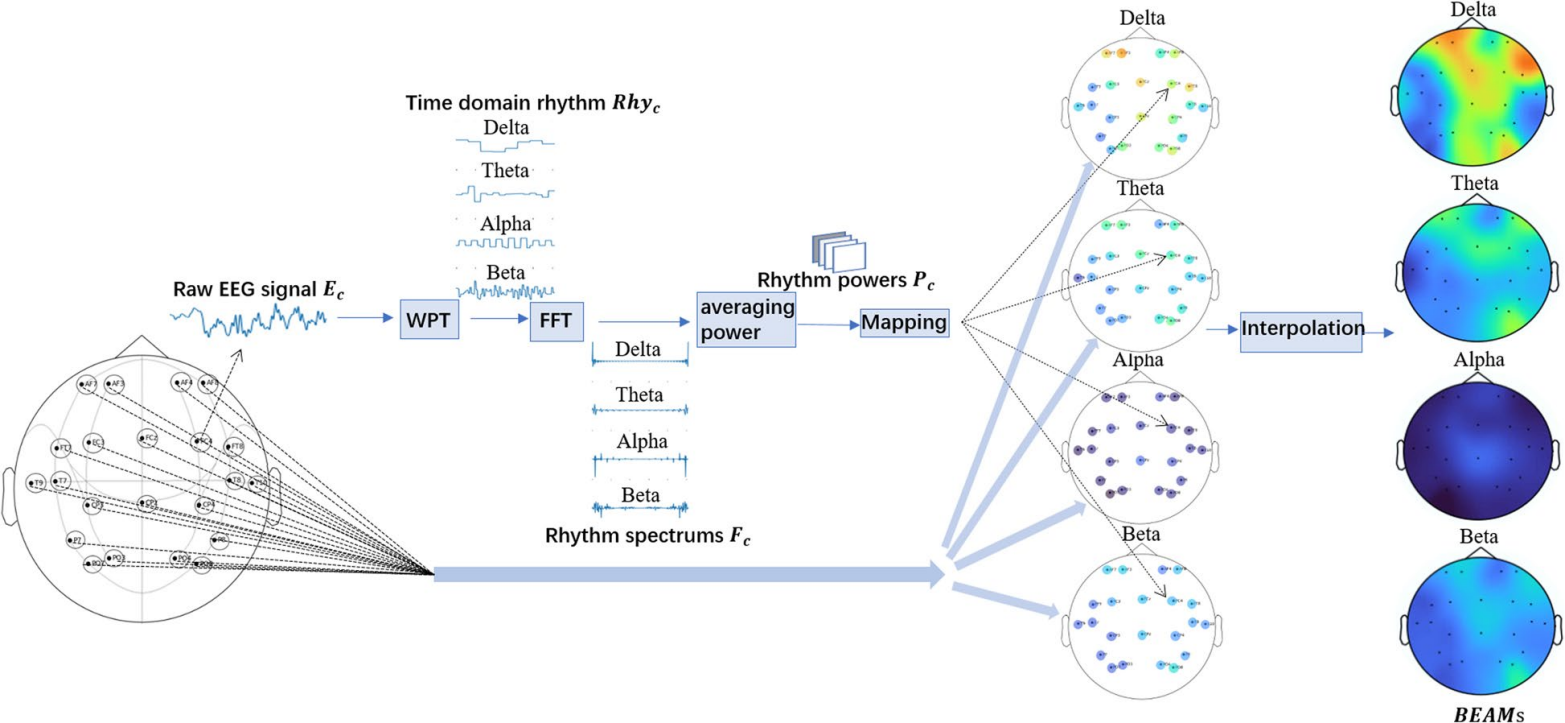
Converting the EEG signal of a single time slice to BEAMs

Figure 3 illustrates the progress of conversion from a time slice of EEG data to BEAMs. This paper first extract four fundamental rhythms from the raw EEG signal of each electrode using WPT and transform these four rhythms from time series to frequency series using FFT; Then calculate the average power of each rhythm; Finally, map the average powers of each rhythm at all electrodes into a two-dimensional head-shaped space and give each point of this space a value by interpolation. The matrix that stores the distribution of the power value of a specific rhythm in the head-shaped space is a BEAM. The progress will be described in detail (for the simplicity of expression, the time index for the time slice of EEG data in the process during the process has been omitted) in the following.

A Extracting time-domain and frequency-domain rhythms

WPT [34] is used to extract specified time-domain rhythms, ${Rhy}_{c}^{b},b=\mathrm{1,2},\dots ,B$ (in the paper they are signals in delta, theta, alpha, beta band respectively), from ${E}_{c}\in {\mathbb{R}}^{S\times 1}$ which is a time slice of the EEG signal from the $c$ th electrode, as follows,

1 $${A}_{c}=WPT\left(wavelet=db1{,\#layers=8,\mathrm{signal}=E}_{c}^{ }\right),c=\mathrm{1,2},\dots ,C$$

2 $${Rhy}_{c}^{b}=IWPT\left(wavelet=db1,\mathrm{ coefficients}=filter({A}_{c},b)\right),b=\mathrm{1,2},\dots ,B$$

where, WPT is the forward WPT which decomposes a time-domain signal into a set of wavelet coefficients, and IWPT is the inverse WPT which reconstructs a time-domain signal from a set of wavelet coefficients. This paper use db1 wavelet function and $\#layers=8$ for the WPT. Function $filter({A}_{c},b)$ let all wavelet coefficients in ${A}_{c}$ zero but those presenting the rhythm b.

FFT is used to extract frequency-domain rhythms ${F}_{c}^{b},b=\mathrm{1,2},\dots ,B$,from the time-domain rhythms${Rhy}_{c}^{b},b=\mathrm{1,2},\dots ,B$, as follows,

3 $${F}_{c}^{b}=FFT\left({Rhy}_{c}^{b}\right)$$

B Calculating rhythm power

The power of the $b$ th rhythm of the $c$ th electrode, ${P}_{c}^{b}$, is calculated from the frequency-domain rhythm ${F}_{c}^{b}$ as,

4 $${P}_{c}^{b}=\frac{{\sum }_{i}^{S}{\left|{F}_{c}^{b}(i)\right|}^{ }}{S} c=\mathrm{1,2},\dots ,C;b=\mathrm{1,2},\dots ,B$$

where S is the number of elements in ${F}_{c}^{b}$. Note that the rhythm powers from C electrodes, B bands and T time slices compose a rhythm power array ${\varvec{P}}\in {\mathbb{R}}^{C\times B\times T}$.

C Getting BEAMs

Let ${{\varvec{L}}}^{3{\varvec{d}}}=[{l}_{1}^{3d},{l}_{2}^{3d},\dots ,{l}_{C}^{3d}]$ be the 3-D locations of the *C* electrodes on a head modeled with an sphere ([35], ${r}^{2}={x}^{2}+{y}^{2}+{z}^{2},r=0.095(m)$), ${{\varvec{L}}}^{2{\varvec{d}}}=[{l}_{1}^{2d},{l}_{2}^{2d},\dots ,{l}_{C}^{2d}]$ be the 2-D locations of the C electrodes on the 2-D flat head mapped from the 3-D head through equidistant azimuthal projection which preserves the distance and direction from any point of the sphere to the center of projection, and ${{\varvec{P}}}^{b}=\left[{{P}_{1}^{b},P}_{2}^{b},\dots ,{P}_{C}^{b}\right]$ be the *C* powers for rhythm *b*. The minimum bounding rectangle of the 2-D head is meshed with equal square unit, getting a grid of size $H\times W$. The 2-D locations of the central points of these squares compose a matrix as,

5 $${{\varvec{L}}}_{{\varvec{g}}{\varvec{r}}{\varvec{i}}{\varvec{d}}}^{2{\varvec{d}}}=\left[\begin{array}{ccc}{l}_{\mathrm{0,0}}^{2d}& \cdots & {l}_{0,W}^{2d}\\ \vdots & \ddots & \vdots \\ {l}_{H,0}^{2d}& \cdots & {l}_{H,W}^{2d}\end{array}\right]$$

The power values of rhythm *b* at these locations compose a power matrix ${BEAM}_{ }^{b}$ of size $H\times W$. For each location $(h,w)$, the corresponding power value in ${BEAM}_{ }^{b}$ is calculated as,

6 $${BEAM}_{ }^{b }(h,w)=Interpolate\left({l}_{h,w}^{2d}|{{\varvec{P}}}^{b},{{\varvec{L}}}^{2d}\right),b=\mathrm{1,2},\dots ,B$$

where, $Interpolate$ is any interpolate function that estimate the value in location $(h,w)$ from existing values ${{\varvec{P}}}^{b}$ and their locations ${{\varvec{L}}}^{2d}$. Here, is used the cubic spline interpolation [36] that satisfies the requirement of smoothness and minimum curvature at the nodes.

#### Generating perturbation on rhythm power array

In this section, from an input $BEAM\in {\mathbb{R}}^{T\times B\times H\times W}$, the progress of getting the perturbation on BEAMs ${\upeta }_{BEAM}\in {\mathbb{R}}^{T\times B\times H\times W}$ and the perturbation on rhythm power array ${\eta }_{{\varvec{P}}}\in {\mathbb{R}}^{T\times B\times C}$ will be described.

A． Getting perturbation on BEAMs

Any known adversarial sample generation algorithm which could deals with a tensor $x\in {\mathbb{R}}^{T\times B\times H\times W}$ as an input could be used in the method in this paper to generate the perturbation on BEAMs, so the perturbation generation algorithm of BEAM is not the focus of this paper. Here, the fast gradient sign algorithm (FGSM) [8] is used for its simplicity to generate the perturbation on BEAMs, as,

7 $${\eta }_{BEAM}= \epsilon sign\left({\nabla }_{BEAM}J\left({\theta }_{victim},BEAM,{y}_{true}\right)\right)$$

where $\epsilon$ is a multiplier to ensure the perturbations are small; $sign$ is the sign function; ${\theta }_{victim}$ are parameters of the victim model; ${y}_{true}$ is the true category of the input tensor $BEAM$; $\mathrm{J}(\uptheta ,BEAM,{\mathrm{y}}_{\mathrm{true}})$ is the loss function. ${\nabla }_{BEAM}\mathrm{J}(\uptheta ,BEAM,{\mathrm{y}}_{\mathrm{true}})$ is the gradient of the corresponding loss function.

B． Getting perturbation on rhythm power array

Let ${\upeta }_{P}\in {\mathbb{R}}^{T\times B\times C}$ denote the perturbation on rhythm power array. For each *t* and *b*, the *C* elements ${\upeta }_{P}(t,b,:)$, could be sampled simply from the $H\times W$ image ${\upeta }_{BEAM}(t,b,:,:)$, according to the 2-D locations of electrodes${{\varvec{L}}}^{2{\varvec{d}}}$, as,

8 $${\upeta }_{P}\left(t,b,c\right)=Interpolate\left({{\varvec{L}}}^{2{\varvec{d}}}\left(c\right)|{\upeta }_{BEAM}\left(t,b,:,:\right),{{\varvec{L}}}_{{\varvec{g}}{\varvec{r}}{\varvec{i}}{\varvec{d}}}^{2{\varvec{d}}}\right),c=\mathrm{1,2},\dots ,C$$

where, $Interpolate$ is any interpolate function that estimate the value in location ${{\varvec{L}}}^{2{\varvec{d}}}\left(c\right)$ from existing values ${\upeta }_{BEAM}\left(t,b,:,:\right)$ and their locations ${{\varvec{L}}}_{{\varvec{g}}{\varvec{r}}{\varvec{i}}{\varvec{d}}}^{2{\varvec{d}}}$*.* Here, cubic spline interpolation [36] is used.

#### Generating EEG adversarial samples

Here, the adversarial samples in frequency domain and time domain are generated based on ${\eta }_{P},$ the perturbation on rhythm power array.

A Imposing perturbation on frequency-domain rhythms

This paper adds the power perturbation ${\upeta }_{P}\left(t,b,c\right)$ on ${F}_{c}^{t,b}\in {\mathbb{C}}^{S}$ which is the raw frequency-domain data of the $b$ th rhythm of the $c$ th electrode and in the $t$ th time slice

9 $$\begin{array}{c}\begin{array}{c}{D}_{c}^{t,b}\left(s\right)=\left(sign\left({F}_{c,R}^{t,b}\left(s\right)\right)*{D}_{c,R}^{t,b}\left(s\right)\right)+\left(sign\left({F}_{c,I}^{t,b}\left(s\right)\right)*{D}_{c,I}^{t,b}\left(s\right)\right)*i\\ {{D}_{c,R}^{t,b}(s)}={{F}_{c,R}^{t,b}(s)}+sign({\eta }_{P}(t,b,c))*{{\eta }_{P}(t,b,c)}^{2}*\frac{{{F}_{c,R}^{t,b}(s)}^{2}}{{{F}_{c,R}^{t,b}(s)}^{2}+ {{F}_{c,I}^{t,b}(s)}^{2}} \\ {{D}_{c,I}^{t,b}\left(s\right)}={{F}_{c,I}^{t,b}\left(s\right)}+sign({\eta }_{P}(t,b,c))*{{\eta }_{P}(t,b,c)}^{2}*\frac{{{F}_{c,I}^{t,b}\left(s\right)}^{2}}{{{F}_{c,R}^{t,b}\left(s\right)}^{2}+ {{F}_{c,I}^{t,b}\left(s\right)}^{2}} \end{array}\\ s=\mathrm{1,2},\dots ,S ,t=\mathrm{1,2},\dots ,T ,b=\mathrm{1,2},\dots ,B;c=\mathrm{1,2},\dots ,C\end{array}$$

where, ${F}_{c,R}^{t,b}$ and ${F}_{c,I}^{t,b}$ denote the real and imaginary parts of the original frequency-domain data of the $b$ th rhythm of the $c$ th electrode and in the $t$ th time slice; ${\eta }_{P}(t,b,c)$ is the power perturbation supposed to be imposed on the $b$ th rhythm of the $c$ th electrode and in the $t$ th time slice. Note that the reconstruction will be done in each dimension of $t,b,c$ and s and finally get a new frequency-domain adversaria data ${\varvec{D}}\in {\mathbb{C}}^{T\times B\times C\times S}$.

B Reconstructing EEG time-domain signal

Here, from the new frequency-domain data*** D*** and the raw time-domain data ${\varvec{E}}$, IFFT and WPT are used to generate the adversarial sample in time-domain, ***É*** The adversarial time signal of the $c$ th electrode in the $t$ th time slice, ${\acute{E} }_{c}^{t}$, are calculated as,

10 $$\begin{array}{c}\begin{array}{c}{\acute{E} }_{c}^{t}=IWPT\left(wavelet=db1,\mathrm{ coefficients}=filter2({A}_{c}^{t},{\acute{A} }_{c}^{t,b},b=[\mathrm{1,2},\dots ,B])\right)\\ {\mathrm{\acute{A} }}_{c}^{t,b}=WPT\left(wavelet=db1,\#layers=8,\mathrm{signal}=\mathrm{IFFT}({{\varvec{D}}}_{c}^{t,b})\right) \end{array}\\ {A}_{c}^{t}=WPT\left(wavelet=db1,\#layers=8,\mathrm{signal}={E}_{c}^{t}\right) t=\mathrm{1,2},\dots ,T;c=\mathrm{1,2},\dots , C\end{array}$$

where function $filter2({A}_{c}^{t},{\mathrm{\acute{A} }}_{c}^{t,b},b=[\mathrm{1,2},\dots ,B])$ replaces the wavelet coefficients presenting rhythms ($b=\mathrm{1,2},\dots ,B$) in ${A}_{c}^{t}$ with corresponding coefficients in ${\acute{A} }_{c}^{t}$ and returns the changed ${A}_{c}^{t}$.

### GPBEAM-DE

**Fig. 4:**
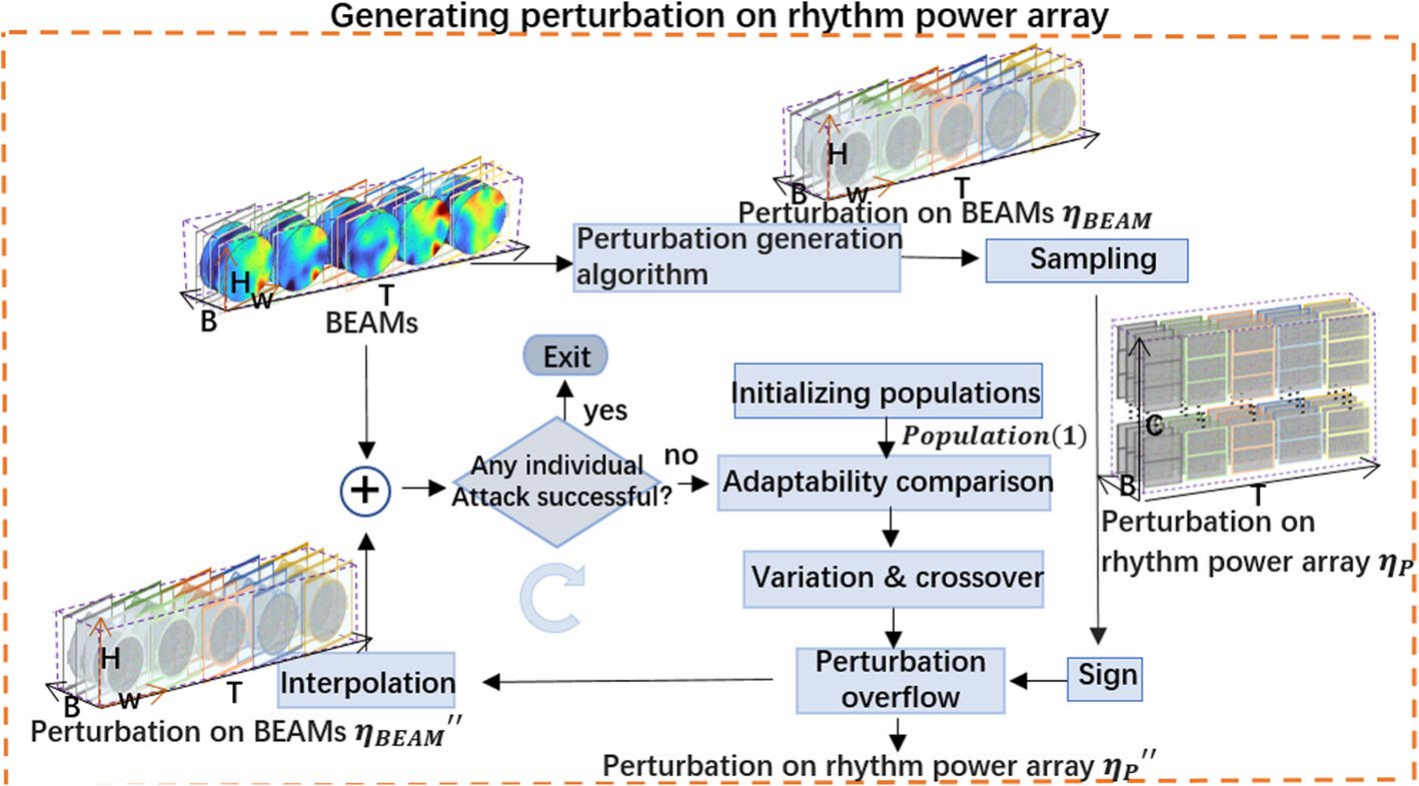
The part of generating perturbation on rhythm power array in GPBEAM-DE

GPBEAM loses some perturbation in the process of sampling perturbation on rhythm power array from perturbation on BEAMs, reducing the aggressiveness of final adversarial samples. The only difference between GPBREAM-DE and GPBEAM is in the part of generating perturbation on rhythm power array (see Fig. 4). In GPBEAM-DE, DE is used to directly perturb partial elements of the rhythmic power array, resulting in more aggressive and sparse adversarial samples. In order to increase the efficiency of DE and to make perturbation imperceptible, a perturbation overflow module is added, in which, when the amplitude of disturbance generated by DE is over a pre-defined level, the excess part will be distributed to other electrodes with the help of the symbolic information of GPBEAM's perturbation.

#### Generating Perturbation with DE

It is set that there are total *NP* individuals in the $g$ th generation of population, with everyone having *N* genes. Each gene is a (2 + *B*)-length integer vector, which represents $\eta \left(t,c,:\right)$, a sparse perturbation of *B* rhythm power values on the *t* time slice and *c* electrode, with first two elements as *t* and *c* and following *B* elements as perturbation of power values. The valid range for *t* is [0, T], for *c* is [0, C], and for perturbation of power values is $[-round(\epsilon *r), round(\epsilon *r)]$, where $\epsilon$ is the same parameter as in Eq. (7) and *r* is an amplification parameter to make $round(\epsilon *r)$ a big integer. When performing a fitness comparison or finally outputting perturbation, the perturbation value is divided by r to get back a real number that is small enough. The goal in this paper is, through DE, to find a perturbation/individual that could successfully attack the victim model and keep the change as small as possible.

The initial population of DE is generated randomly and uniformly as follow,

11 $$\begin{array}{c}Population(g=1)=\left\{{X}_{1}\left(g\right),{X}_{2}\left(g\right),\dots ,{X}_{NP}\left(g\right)\right\}\\ {X}_{i}\left(g\right)=\left[{gene}_{1},{gene}_{2},\dots ,{gene}_{N}\right],i=\mathrm{1,2},\dots ,NP\\ \begin{array}{c}{gene}_{n}=\left[t,c,{v}_{n}\left(1\right),{v}_{n}\left(2\right),\dots ,{v}_{n}(B)\right],n=\mathrm{1,2},\dots ,N\\ t=rand\_int\left(1,T\right)\\ \begin{array}{c}c=rand\_int\left(1,C\right)\\ v\left(b\right)=rand\_int\left(-round(\epsilon *r), round(\epsilon *r)\right), b=\mathrm{1,2},\dots B\end{array}\end{array}\end{array}$$

where, $rand\_int()$ randomly samples an integer from the input interval.

In each iteration of evolution, the offspring individuals are produced through mutation and crossover, as,

12 $$\begin{array}{c}{X}_{i}\left(g+1\right)=\left\{\begin{array}{c}{U}_{i}\left(g+1\right)\ if\ fitness\left({U}_{i}\left(g+1\right)\right)>fitness({X}_{i}\left(g\right)) \\ {X}_{i}\left(g\right) otherwise\end{array}\right.i=\mathrm{1,2},\dots ,NP\\ {U}_{i,j}\left(g+1\right)= \left\{\begin{array}{c}{V}_{i,j}\left(g+1\right) if\ rand\left(\mathrm{0,1}\right)\le CR\\ {x}_{i,j}\left(g\right) otherwise\end{array}\right.,j=\mathrm{1,2},\dots ,N\\ {V}_{i}\left(g+1\right)=valid\_int\left({X}_{r1}\left(g\right)+F\left({X}_{r2}\left(g\right)-{X}_{r3}\left(g\right)\right)\right)\end{array}$$

where $r1$, $r2$, $r3$ are three different indexes randomly selected from $\left\{\mathrm{1,2},\dots ,NP\right\}$; $F\in \left[\mathrm{0,2}\right]$ is a scaling real factor; $CR\in \left[\mathrm{0,1}\right]$ is a crossover probability; $rand(\mathrm{0,1})$ produces a uniformly distributed random real from $\left[\mathrm{0,1}\right]$; $valid\_int(X)$ makes all elements of genes of the individual *X* integers by rounding, and if any integer exceeds its valid range, produces a valid random number to replace it.

${U}_{i}(g+1)$ need to compete with its corresponding parent candidate ${X}_{i}\left(g\right)$ according to the fitness, and the winner is kept until the next iteration. The fitness measure, with ${\mathrm{X}}_{i}\left(g\right)$ as input for example, is defined as,

13 $$\begin{array}{c}\mathrm{fitness}\left({\mathrm{X}}_{\mathrm{i}}\left(\mathrm{g}\right)\right)=1-{\mathrm{P}}_{\mathrm{victim}}(\mathrm{y}={\mathrm{y}}^{*}|{\mathrm{map}}_{\mathrm{P}\to \mathrm{BEAM}}({{\varvec{\eta}}}_{\mathbf{P}}^{\mathrm{^{\prime}}}+\mathbf{P}))\\ {y}^{*}={argmax}_{y\in Y}({\mathrm{P}}_{\mathrm{victim}}(y|{\mathrm{map}}_{\mathrm{P}\to \mathrm{BEAM}}({{\varvec{\eta}}}_{{\varvec{P}}}^{\boldsymbol{^{\prime}}}+\mathbf{P})))\end{array}$$

where ${{\varvec{\upeta}}}_{{\varvec{P}}}^{\mathbf{^{\prime}}}\in {\mathbb{R}}^{T\times B\times C}$ is created from ${X}_{i}\left(g\right),$ with all its elements zeros but those defined by genes of ${X}_{i}\left(g\right)$; ${\mathrm{map}}_{\mathrm{P}\to \mathrm{BEAM}}\left({{\varvec{\upeta}}}_{{\varvec{P}}}^{\mathrm{^{\prime}}}+\mathbf{P}\right)$ adds the perturbation of ${{\varvec{\upeta}}}_{{\varvec{P}}}^{\mathrm{^{\prime}}}$ to ***P*** and then converts the resulted ***P*** into BEAMs; ${P}_{\mathrm{victim}}\left(y|BEAMs\right)$ returns from victim model the prediction probability that the input BEAMs belong to category y.

The iteration of DE ends, when any individual of the population (g + 1) matches the following formula,

14 $${y}^{*}\ne {argmax}_{y\in Y}(P(y|{map}_{P\to BEAM}({{\varvec{\upeta}}}_{{\varvec{P}}}^{\mathrm{^{\prime}}}+\mathbf{P})))$$

#### Perturbation overflow

**Fig. 5:**
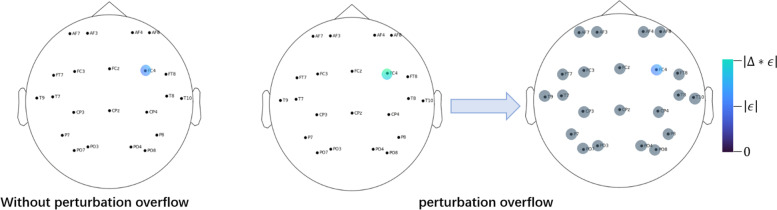
In GPBEAM-DE without perturbation overflow, the perturbation added on one electrode are limited below $\epsilon$ (left). In GPBEAM-DE with Perturbation overflow, the perturbation added on one electrode could be a little bigger at first (middle), but then the perturbation over $\epsilon$ is distributed equally to all other electrodes (right)

Perturbation overflow is a step in GPBEAM-DE, which increases the attack power of adversarial samples from GPBEAM-DE by decreasing the sparsity of their attacks in a very natural way (see Fig. 5) of distributing the excess perturbations on a few sparse electrodes equally to all other electrodes. By adding perturbation overflow to GPBEAM-DE, the efficiency of generating successful adversarial samples improves.

In order to use perturbation overflow in GPBEAM-DE, the valid range for perturbation of power value should be expanded a little bit with $$\Delta =C$$ as $$[-round(\epsilon *r*\Delta ), round(\epsilon *r*\Delta )]$$. Then the only thing that perturbation overflow do is to replace each $${\upeta }_{P}^{\mathrm{^{\prime}}}$$ that generated from $${X}_{i}\left(g\right)$$ in Eq. (13) with a new perturbation $${{\upeta }_{P}}^{^{\prime\prime} }$$. The new perturbation is generated as,

15 $$\begin{array}{c}{{\upeta }_{P}\left(t,b,:\right)}^{^{\prime\prime} }=clip\left(\frac{{\sum }_{t=1}^{T}{\sum }_{b=1}^{B}{\sum }_{c=1}^{C}1\left(\left|{{\upeta }_{P}\left(t,b,c\right)}^{\mathrm{^{\prime}}}\right|>\epsilon \right)*\left(\left|{{\upeta }_{P}\left(t,b,c\right)}^{\mathrm{^{\prime}}}\right|-\epsilon \right)}{C}*sign\left({\upeta }_{P}\left(t,b,:\right)\right)+{{\upeta }_{P}\left(t,b,:\right)}^{\mathrm{^{\prime}}},-{\epsilon }_{ },{\epsilon }_{ }\right)\\ t=\mathrm{1,2},\dots ,T;b=\mathrm{1,2},\dots ,B\end{array}$$

where, $clip$ is a crop function; $1\left(condition\right)$ return 1, if condition is True, else return 0; ${\upeta }_{P}$ is the perturbation generated by GPBEAM (see Eq. 7). $sign\left({\upeta }_{P}\left(t,b,:\right)\right)$ is used to extract symbolic information of ${\upeta }_{P}\left(t,b,:\right)$. Ultimately, GPBEAM-DE will have the advantages of both DE and GPBEAM.

## Experiments and analysis

### Description of experimental data

The experimental data, the CHB-MIT Scalp EEN Database [37, 38], was collected from Boston Children's Hospital and included EEG records of 22 children with recalcitrant epilepsy. Subjects were monitored for up to several days after discontinuation of antiepileptic drugs to characterize their seizures. Experiments were performed using the international 10–20 standard for laying out EEG electrode positions. All EEG signals were sampled at a sampling rate of 256 Hz. EEG signals have 23 channels, of which only 22 are used here. In addition, to facilitate the reconfiguration of the EEG into BEAMs [39], the channel names in the CHB-MIT scalp EEG database are corresponded to those of the international 10–20 standard.

This paper gets a total of 7016 raw EEG samples, by firstly tailoring the experimental data to a series of 5 s-length segments (2 s-overlapping for seizures and non-overlapping for non-seizures), and then selecting all seizure segments and the equal number of non-seizure segments. The final size of the raw EEG sample or the EEG adversarial sample is 5(time slice) * 22 (electrodes) * 256 (number of samples per second). Bad data are deleted and data are normalized before tailoring. Of all the raw EEG samples, 5612 are used for training the seizures detection models (victim models), and 1404 for generating adversarial samples. Subsequent experiments were conducted on this premise.

As shown in Fig. 3, one time slice of raw EEG signal can be reconstructed into four BEAMs, of which each represents an EEG rhythm. By setting the length of a time slice to be one second, a BEAMs sample of size 5 (time slice) * 4 (rhythm) * 22 (length) * 22 (width) will be got from each raw EEG sample. The information of the dataset used in this article is summarized in Table 2.

**Table 2:** Information sheet for dataset used in this paper

	non-seizures	seizures
number of samples (Training set)	2806	2806
number of samples (Test set)	702	702
overlaps of time slices	non-overlapping	2 s-overlapping
size of each EEG sample	5(time slice) * 22 (electrodes) * 256 (number of samples per second)	
size of each BEAMs sample	5 (time slice) * 4 (rhythm) * 22 (length) * 22 (width)	

### Victim models

**Fig. 6:**
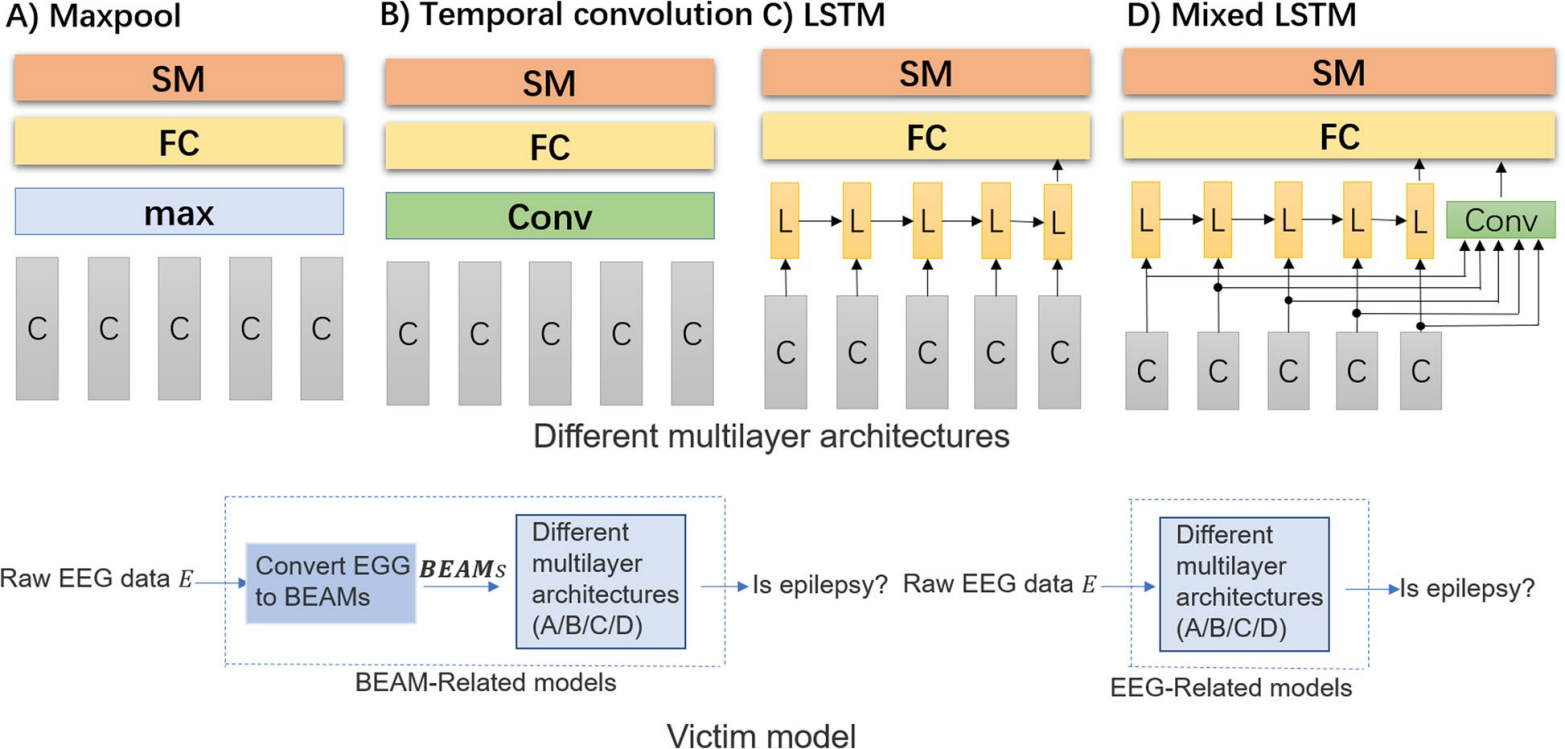
Different multilayer architectures and Victim models; C: ConvNet; Max: maximum pooling layer; FC: fully connected layer; SM: softmax layer; Conv: 2D convolutional layer; L: LSTM layer

Two types of victim models, the BEAM-related model and the EEG-related model, were used. They use the same inputs of EEG data and similar multilayer architectures (see Fig. 6). The main difference between them is that the first type needs to extract BEAMs features and then let them pass through multilayer architectures, and the second type directly passes EEG data through multilayer architectures.

Four multi-layer architectures proposed by Bashivan et al. [7] are used. Maxpool and Temporal convolution are pure CNN architectures, LSTM and Mixed LSTM are CNN + RNN architectures.

The number of parameters of the fully connected layer and the number of LSTMs in multilayer architectures differs a little bit from those used by Pouya Bashivan et al. because the inputs used are different. The ConvNet configurations of victim models is described in Table 3.

**Table 3:** ConvNet configurations

victim models	BEAM-related models	EEG-related models
Input	22*22 4-channel	22*256 1-channel
Convolution + ReLU	3*3*32	
Convolution + ReLU	3*3*32	
Convolution + ReLU	3*3*32	
Convolution + ReLU	3*3*32	
Max Pooling	2*2	
Convolution + ReLU	3*3*64	
Convolution + ReLU	3*3*64	
Max Pooling	2*2	
Convolution + ReLU	3*3*128	
Max Pooling	2*2	

Model training is carried out by optimizing the cross-entropy loss function. The networks are trained using Adam algorithm with a learning factor of ${10}^{-3}$, and decay rate of first and second moments as 0.9 and 0.999 respectively. In the experiments, only the EEG-related model with Mixed LSTM architecture suffered from overfitting. The complexity of Mixed LSTM architecture is higher compared to that of other architectures, which should be the cause of overfitting. In this paper, L2 regularization, Dropout (dropout probability is set to 0.5, i.e., the network discards neurons with a probability of 0.5) and adjusting learning rate are used to reduce overfitting of this model.

In the end of training, the test accuracies of BEAM-related models with Maxpool, Temporal convolution, LSTM and Mixed LSTM were 92%, 92%, 93%, and 94%. The training losses were all less than ${10}^{-3}$ and the test losses were 0.63, 0.55, 0.27, and 0.63, respectively.

**Fig. 7:**
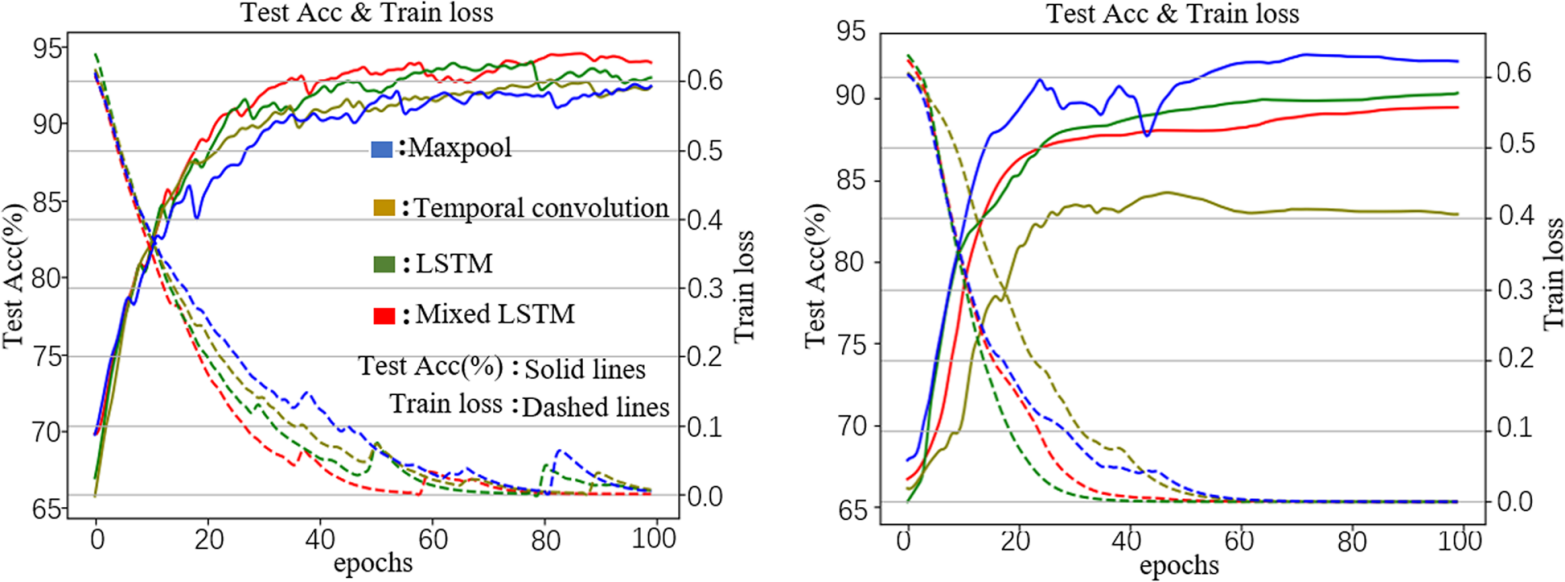
Training loss and test accuracy curves for BEAM-related models (left) and EEG-related models (right)

In the end of training, the test accuracy of EEG-related models with Maxpool, Temporal convolution, LSTM and Mixed LSTM were 92%, 84%, 90%, and 88%. The training losses were all less than ${10}^{-4}$ and the test losses were 0.38, 0.79, 0.50, and 0.54, respectively. More information can be found in Fig. 7.

### Evaluation criteria

In this paper, the performance of GPBEAM/GPEBAM-DE are evaluated with three metrics.

- **Success rate (SR)**: As one of the most important measures for adversarial attack, it indicates the percentage of adversarial samples that successfully change their raw predicted labels. A higher SR indicates that the algorithm is more capable of attacking, and means that the target classifier is more vulnerable to attack.
  16 $$SR = R/S$$

where S is the total number of samples and R is the number of samples that succeeded in the attack.

b) **Distortion level (DL)**: It is used to measure the distortion of adversarial samples relative to the raw samples. ${\mathrm{DL}}_{B}$ and ${\mathrm{DL}}_{E}$ are used to indicates the distortion of BEAMs adversarial samples and EEG adversarial samples respectively. They are defined as follows,where $N$ and $M$ are the number of elements of a BEAMs sample and an EEG sample, respectively; ${\widehat{B}}_{n}^{s}$ and ${B}_{n}^{s}$ are the *n*th element of the BEAMs adversarial sample and BEAMs raw sample respectively; ${\widehat{E}}_{m}^{s}$ and ${E}_{m}^{s}$ are the $m$ th element of the EEG adversarial sample and the EEG raw sample respectively.
  17 $${\mathrm{DL}}_{B}=\frac{{\sum }_{s=1}^{S}\sqrt{\frac{{\sum }_{n=1}^{N}{\left({\widehat{B}}_{n}^{s}-{B}_{n}^{s}\right)}^{2}}{N}}}{S}$$
  18 $${\mathrm{DL}}_E=\frac{\sum_{s=1}^S\sqrt{\frac{\sum_{m=1}^M\left(\widehat E_m^s-E_m^s\right)^2}M}}S.$$

iii) **Accuracy (Acc)**: It measures the probability that a victim model predicts correctly and is defined,where A is the number of samples that the model classifies correctly.
  19 $$Acc = A/S$$

### Experiment 1: attacking BEAM-related models with GPBEAM

First, the aggression of GPBEAM to BEAM-related models with different multilayer architectures is tested. In this experiment, FGSM [8] is chosen as the perturbation generation method of GPBEAM. FGSM is less aggressive than other state-of-arts methods. If GPBEAM with FGSM can successfully attack BEAM-related models, then GPBEAM with other perturbation methods can naturally attack successfully.

**Fig. 8:**
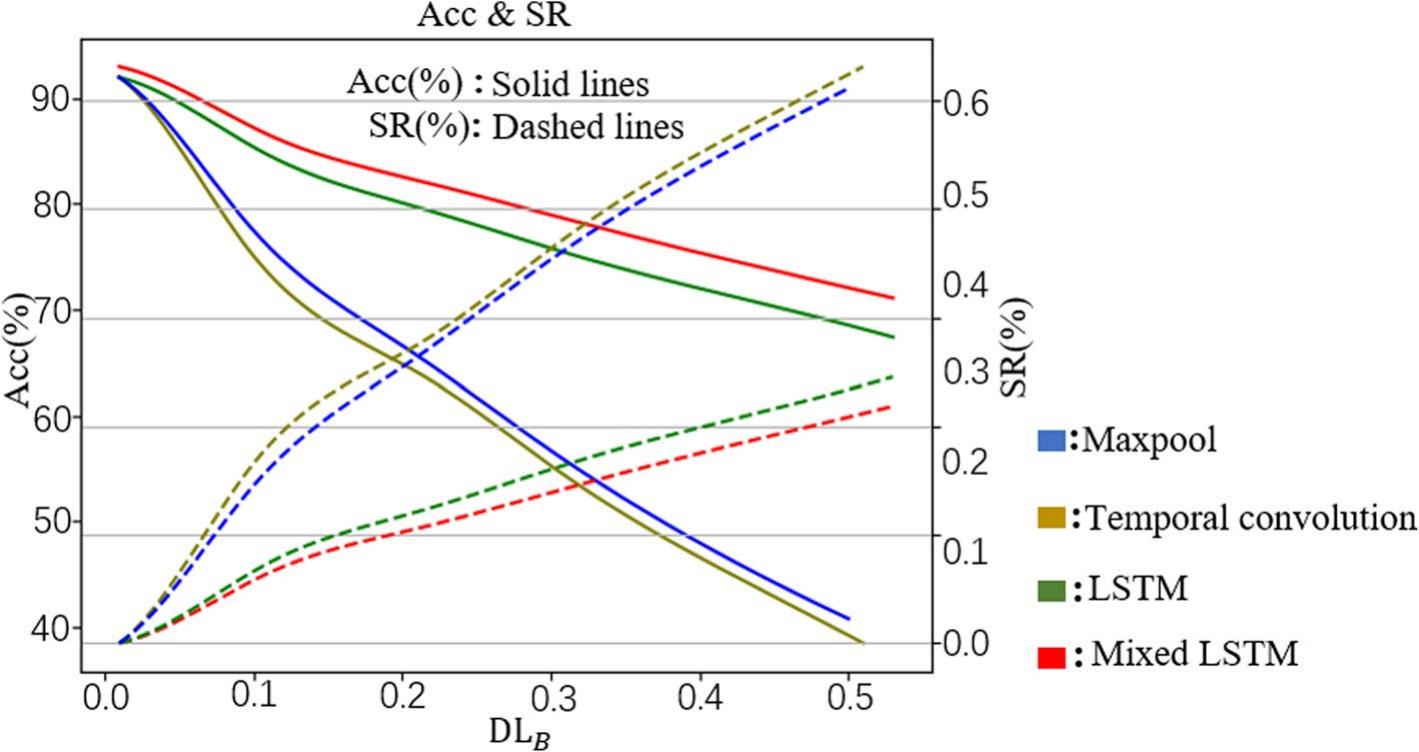
Acc vs ${{\varvec{D}}{\varvec{L}}}_{{\varvec{B}}}$ and SR vs ${{\varvec{D}}{\varvec{L}}}_{{\varvec{B}}}$ for BEAM-related models with four different architectures (each Acc/SR value is got with 1404 test trials)

As shown in Table 4**,** BEAM-related models with different multi-layer architectures can achieve more than 90% accuracy when classifying clean data. However, after adding negligible perturbation to the clean data, the classification accuracy of these victim models decreases significantly. Compared with Gaussian noise, the attack effect of GPBEAM is obvious. As shown in Fig. 8, the accuracy of BEAM-related models with pure CNN architectures (Maxpool and Temporal convolution) decreases particularly significantly as ${\mathrm{DL}}_{B}$ increases. From the attack success rate (SR) curves, GPBEAM attacks BEAM-related models with pure CNN architectures have higher success rates than attacks on BEAM-related models with CNN + RNN architectures. BEAM-related models with CNN + RNN architecture are more robust to GPBEAM attacks than the BEAM-related models with CNN architecture, as seen from the above experiments. It is suspected that BEAMs are richer in spatial features than temporal and frequency features, and GPBEAM mainly perturbs spatial features. This makes GPBEAM more aggressive to CNN architectures that mainly extract spatial features and less aggressive to RNN architectures that mainly exploit temporal features.

**Table 4:** GPBEAM attacks BEAM-related models of different multi-layer architectures (ε is a parameter to ensure the perturbations are small. In this table, the maximum value of $\epsilon$ is 0.5, which is much smaller than 50, the maximum value of elements in BEAMs. Adding gaussian noise (Gn) with a mean of 0 and a standard deviation of 0.5 is as the baseline attacking for comparison.)

Architecture	Maxpool			Temporal convolution			LSTM			Mixed LSTM		
$\epsilon$	Acc	SR	${\mathrm{DL}}_{B}$	Acc	SR	${\mathrm{DL}}_{B}$	Acc	SR	${\mathrm{DL}}_{B}$	Acc	SR	${\mathrm{DL}}_{B}$
$0$	0.92	-	-	0.92	-	-	0.93	-	-	0.94	-	-
$0.1$	0.42	0.50	0.10	0.34	**0.57**	0.10	0.68	0.24	0.11	0.72	0.21	0.10
$0.3$	0.17	0.75	0.30	0.15	**0.77**	0.21	0.56	0.36	0.32	0.62	0.31	0.31
$0.5$	0.12	0.80	0.50	0.09	**0.82**	0.51	0.53	0.39	0.53	0.58	0.35	0.52
**Gn** (Baseline)	0.84	0.12	0.69	0.81	0.16	0.69	0.84	0.13	0.69	0.79	**0.17**	0.69

Second, the aggressiveness of GPBEAM with different perturbation generation algorithms are tested. I-FGSM (Iterative-FGSM) [9], MI-FGSM (Momentum iterative- FGSM) [40], DII-FGSM (Diverse Input Iterative-FGSM) [41], PGD (Projected Gradient Descent) [42] and C&W (Carlini & Wagner) [43] are used here as perturbation generation algorithms. The BEAM-related model with a Mixed LSTM architecture is used as the victim model. As shown in Table 5, the attack performance of GPBEAM with these perturbation generation algorithms are obviously better than GPBEAM with FGSM (see Table 4). Among them, GPBEAM (C&W) is the most aggressive.

**Table 5:** Performance of GPBEAM with different perturbation generation algorithms

	I-FGSM			MI-FGSM			DII-FGSM			PGD			C&W		
$\epsilon$	0.1	0.3	0.5	0.1	0.3	0.5	0.1	0.3	0.5	0.1	0.3	0.5	0.1	0.3	0.5
Acc	0.75	0.58	0.50	0.57	0.47	0.43	0.69	0.54	0.46	0.52	0.43	0.41	0.49	0.40	0.34
SR	0.18	0.35	0.43	0.37	0.46	0.52	0.25	0.39	0.47	0.41	0.51	0.53	**0.45**	**0.55**	**0.59**
${\mathrm{DL}}_{B}$	0.09	0.22	0.33	0.10	0.22	0.35	0.09	0.21	0.34	0.08	0.23	0.37	0.10	0.26	0.45

**Fig. 9:**
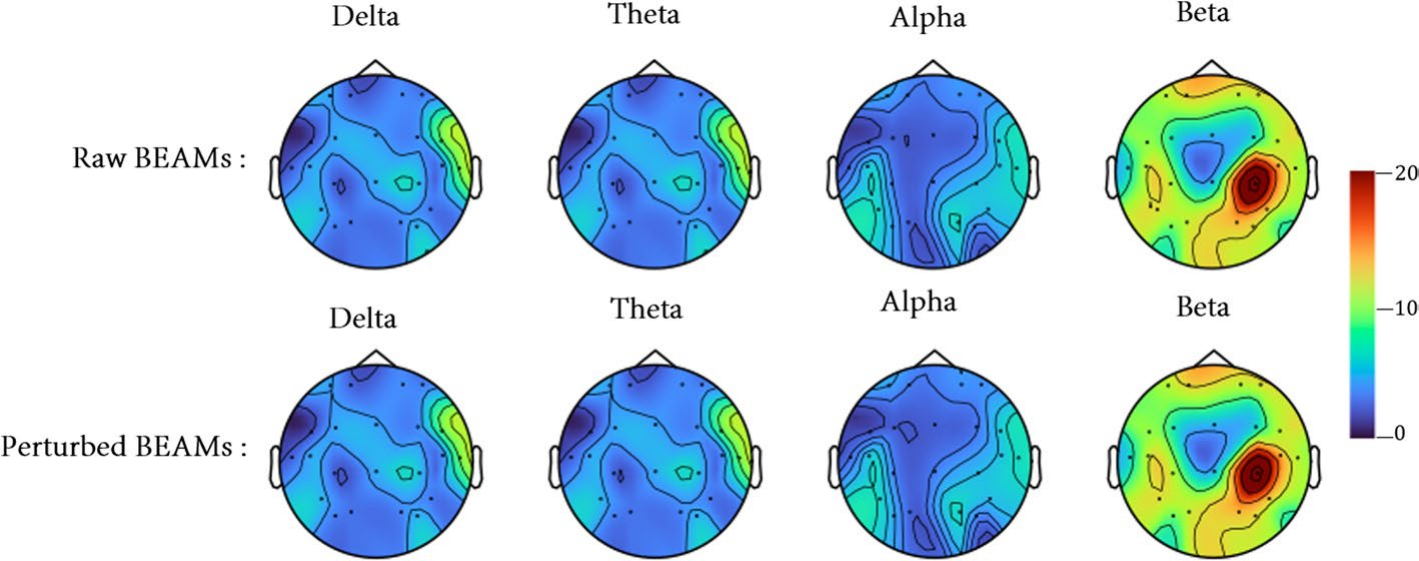
Comparison of the perturbed BEAMs and the raw BEAMs

Figure 9 shows a comparison of a perturbed BEAMs sample (generated by GPBEAM with $\epsilon$=0.5 and FGSM as perturbation generation method) and the corresponding raw BEAMs sample. The final perturbations of BEAMs do not exhibit the characteristics of random noise and it is almost impossible for the naked eye to immediately distinguish between the perturbed and raw samples.

**Fig. 10:**
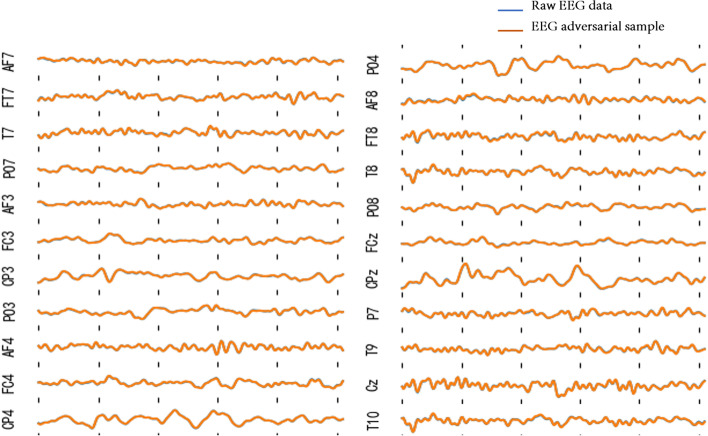
Comparison of the EEG adversarial sample and the raw EEG data of one time slice. The perturbed data (yellow line) overlap with and thus cover the raw EEG data (blue line)

**Fig. 11:**
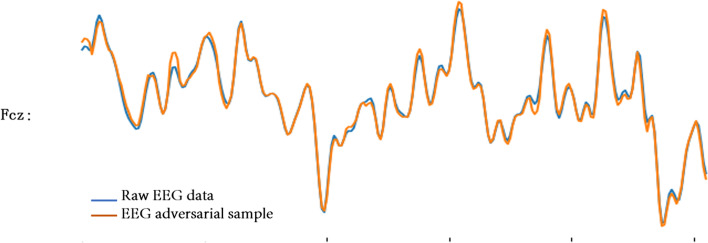
EEG adversarial sample and raw EEG data on Fcz electrode

The differences between a raw EEG data and an EEG adversarial sample (generated by GPBEAM with $\epsilon$=0.5 and FGSM as perturbation generation method) are shown in Fig. 10. The EEG adversarial sample and raw EEG data overlap almost completely and cannot be distinguished by human eyes. As shown in Fig. 11, if the data in Fig. 10 is magnified several times, the difference between the two will show, but they are still extremely similar.

### Experiment 2: attacking BEAM-related models with GPBEAM-DE

In this experiment, it is tested that whether the sparse adversarial samples generated by GPBEAM-DE can effectively attack the BEAM-related models and that whether GPBEAM-DE can achieve a higher attack success rate with less distortion than GPBEAM.

The BEAM-related model with a Mixed LSTM architecture is used as the victim model and FGSM as the perturbation generation method. To test the sparse aggressiveness of GPBEAM-DE, the number of genes N in each-individual of GPBEAM-DE was set to be different values. The experimental results are shown in Table 6. By comparing the results in Table 6 and that in Table 4 and Table 5, it is clear that GPBEAM-DE outperforms GPBEAM in both SR and DL when parameter *N* is bigger than 1. The likely reason for this result is that GPBEAM loses some perturbation in the process of sampling perturbation on rhythm power array from perturbation on BEAMs, reducing the aggressiveness of final adversarial samples, while GPBEAM-DE uses DE to directly perturbate some elements of rhythm power array, resulting in more aggressive and sparser adversarial samples.

**Table 6:** GPBEAM-DE attacking BEAM-related models. GPBEAM-DE-N denote the GPBEAM-DE with N genes in each individual. GPBEAM-DE-5* indicates GPBEAM-DE-5 without perturbation overflow

Attack Method	GPBEAM-DE-5*			GPBEAM-DE-5			GPBEAM-DE-3			GPBEAM-DE-1		
$\epsilon$	0.1	0.3	0.5	0.1	0.3	0.5	0.1	0.3	0.5	0.1	0.3	0.5
Acc	0.93	0.73	0.66	0.64	0.25	0.15	0.81	0.37	0.21	0.89	0.73	0.65
SR	0.02	0.23	0.30	**0.31**	**0.70**	**0.80**	0.23	0.59	0.74	0.05	0.23	0.31
${\mathrm{DL}}_{B}$	0.01	0.04	0.07	0.06	0.18	0.29	0.04	0.12	0.19	0.02	0.05	0.07

To analysis the effect of perturbation overflow, this paper has done ablation experiments of perturbation overflow and the results are shown in Table 6. The aggressiveness of GPBEAM-DE-5 with perturbation overflow is substantially higher than that of GPBEAM-DE-5 without perturbation overflow. Compared to GPBEAM-DE-5 without perturbation overflow, the SR of GPBEAM-DE-5 with perturbation overflow is improved by between 0.3 and 0.5. This exactly meets expectation that perturbation overflow is effective. But it should be noted that the improvement in aggressiveness is got at the expense of higher distortion (the DL is increased by a factor of about 5).

### Experiment 3: the transferability of EEG adversarial samples generated by GPBEAM/GPBEAM-DE

First, the transferability of adversarial samples generated by GPBEAM and GPBEAM-DE among BEAM-related models is tested. Specifically, the BEAM-related model with a Mixed LSTM architecture is used as the source victim model and the BEAM-related models with other architectures as the target victim models. This experiment attacks the source model and apply the resulting adversarial samples to trick the target models. The FGSM is used as the perturbation generation method in GPBEAM and GPBEAM-DE.

The results are shown in Table 7. When both the source and target models are BEAM-related models, the transferability of adversarial samples is obvious with the SR values on target models being still considerable. The SR decreases a maximum of 0.26 when the adversarial samples generated by GPBEAM-DE are transferred, and in contrast, the SR decreases a maximum of 0.05 when the adversarial samples generated by GPBEAM are transferred, indicating that adversarial samples of GPBEAM have better transferability than those of GPBEAM-DE.

**Table 7:** Transferability of adversarial samples when both the source model (with Mixed LSTM architecture) and target models (with other architectures) are BEAM-related models. The N of GPBEAM-DE is 5. Since the adversarial samples are all obtained from the source model, the values of ${DL}_{B}$ or ${DL}_{E}$ are the same for all architectures, and so only the ${DL}_{B}$ and ${DL}_{E}$ of Mixed LSTM (source) are shown here

Architecture		Maxpool		Temporal convolution		LSTM		Mixed LSTM (source)			
$\epsilon$	Evaluation Criteria	Acc	SR	Acc	SR	Acc	SR	Acc	SR	${\mathrm{DL}}_{B}$	${\mathrm{DL}}_{E}$
-	-	0.92	-	0.92	-	0.93	-	0.94	-	-	-
0.1	$\mathrm{GPBEAM}$	0.80	**0.15**	0.79	0.14	0.80	**0.15**	0.73	0.20	0.11	0.024
	GPBEAM-DE	0.83	0.13	0.76	0.18	075	**0.19**	0.64	0.31	0.06	0.021
0.3	$\mathrm{GPBEAM}$	0.67	0.27	0.66	0.28	0.65	**0.29**	0.61	0.32	0.32	0.030
	GPBEAM-DE	0.46	0.48	0.39	**0.52**	0.44	0.49	0.25	0.70	0.18	0.026
0.5	$\mathrm{GPBEAM}$	0.62	0.32	0.60	**0.33**	0.61	0.32	0.56	0.37	0.53	0.036
	GPBEAM-DE	0.36	0.60	0.32	**0.66**	0.35	0.62	0.15	0.80	0.29	0.030

Second, the transferability of the adversarial samples generated by GPBEAM and GPBEAM-DE from BEAM-related models to EEG-related models is tested. Here, the source and target models use the same multilayer architecture. The results are shown in Table 8. When the target model is EEG-related models, the adversarial sample generated by GPBEAM and GPBEAM-DE have almost no aggressiveness. The likely reason is that the process of converting each time slice of EEG to BEAM loses nearly all in-slice information that is very important to those EEG-related models. In addition, GPBEAM-DE is worse than GPBEAM in transferability, and this paper suspect that is due to the sparse perturbation nature of GPBEAM-DE.

**Table 8:** Transferability of the adversarial samples when the source model is BEAM-related model and the target model is EEG-related models. The N of GPBEAM-DE is 5

Architecture			Maxpool		Temporal convolution		LSTM		Mixed LSTM	
${\mathrm{DL}}_{E}$	$\epsilon$	Evaluation Criteria	Acc	SR	Acc	SR	Acc	SR	Acc	SR
0	-	-	0.92	-	0.84	-	0.90	-	0.88	-
0.024	0.1	$\mathrm{GPBEAM}$	0.92	0.01	0.84	0.01	0.90	0.01	0.88	0.01
0.021	0.1	GPBEAM-DE	0.92	0.01	0.84	0.01	0.90	0.01	0.88	0.01
0.030	0.3	$\mathrm{GPBEAM}$	0.92	0.01	0.84	0.02	0.90	0.01	0.88	0.01
0.026	0.3	GPBEAM-DE	0.92	0.01	0.84	0.01	0.90	0.01	0.88	0.01
0.036	0.5	$\mathrm{GPBEAM}$	0.91	**0.02**	0.84	**0.03**	0.90	0.01	0.88	**0.02**
0.030	0.5	GPBEAM-DE	0.92	0.01	0.84	0.01	0.90	0.01	0.88	0.01

In addition, this paper uses the EEG adversarial samples generated by GPBEAM/GPBEAM-DE to attack the frequency-related models (these models are trained by feeding frequency domain representation of EEG signals to multi-layer architectures. FFT is used here for extracting frequency domain representation) and the time–frequency related models [44] (these models are trained by feeding time–frequency domain presentation of EEG data to multi-layer architectures. Wigner-Ville method, one of the methods mentioned in [44] is used here for extracting time–frequency representation). However, the results of both experiments were not satisfactory (the attack success rate is about the same as Gaussian noise).

EEG adversarial samples generated by GPBEAM/GPBEAM-DE cannot attack EEG-related models, frequency-related models and time–frequency-related models just for the same key reason. That is GPBEAM/GPBEAM-DE are white-box methods, for which good performance must be with the right kind of victim models. Because GPBEAM/GPBEAM-DE focus on BEAM-related victim models, their attacks are almost non-aggressive to victim models that is not BEAM-related.

Fortunately, has been found a way (just a simple modification to the methods in this paper) to make it possible that GPBEAM/GPBEAM-DE could also attack victim models that are not BEAM-related. The key idea is that fusing the information of the adversarial sample for attacking BEAM-related models and information of the adversarial samples for attacking other kind of victim models may make the final adversarial sample be aggressive to all these victim models. The details of this modification are in Experiment 4: attacking both BEAM-related and EEG-related models with modified GPBEAM and modified GPBEAM-DE. It should be noted that in the similar way as in experiment 4, the modified GPBEAM/GPBEAM-DE may also attack frequency-related models and other kind of models.

### Experiment 4: attacking both BEAM-related and EEG-related models with modified GPBEAM and modified GPBEAM-DE

The future epilepsy diagnosis models may detect features from raw EEG, BEAMs, or both, considering that the diagnosis of epilepsy requires human doctors to analyze both the raw EEG and BEAMs signals. Therefore, it should be an advantage that the adversarial samples could attack both EEG-related models and the BEAM-related models.

**Fig. 12:**
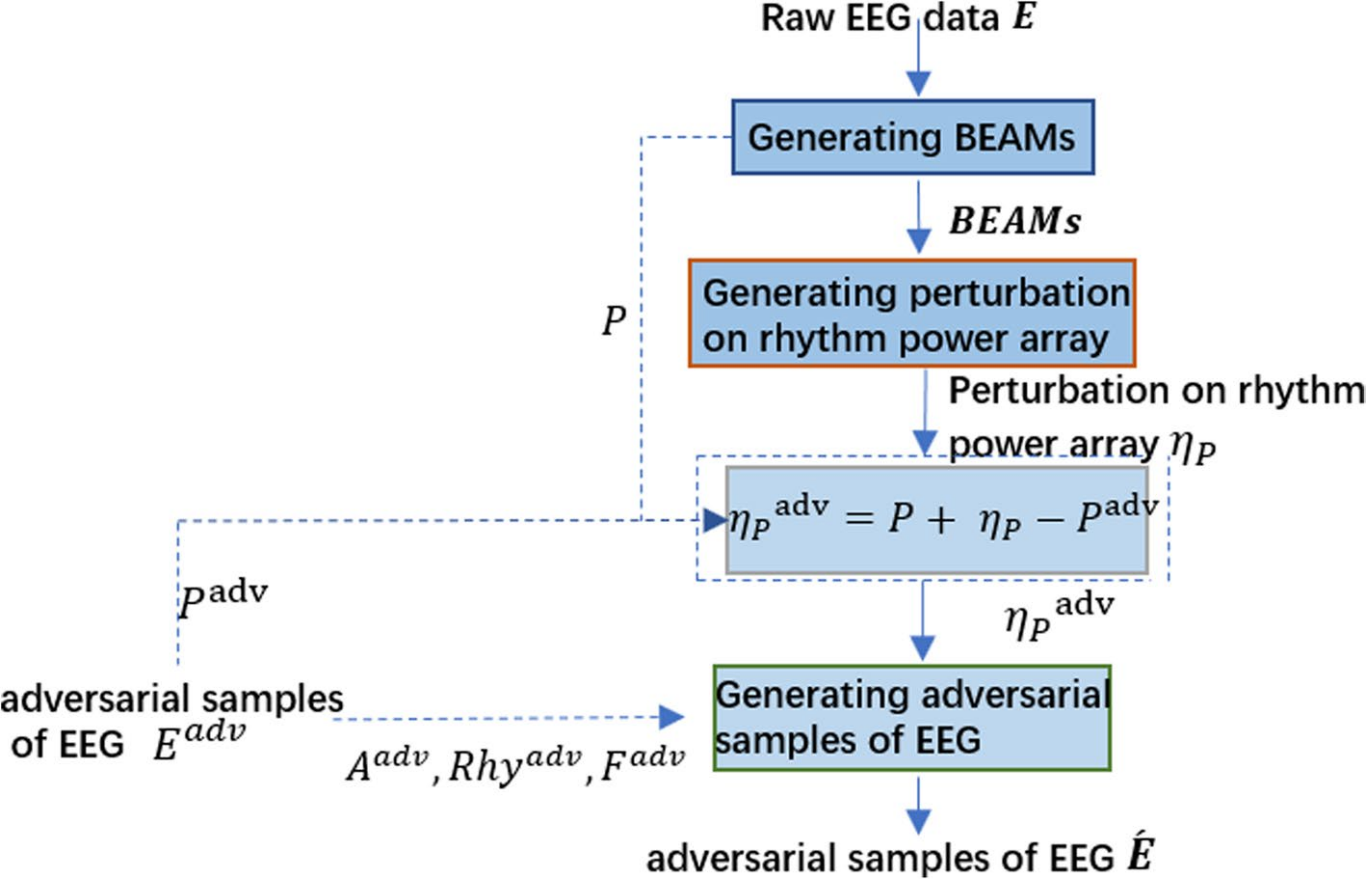
Modified GPBEAM /GPBEAM-DE. The dotted line indicates the modified part

This paper makes a simple modification to the GPBEAM/GPBEAM-DE to make it aggressive to both BEAM-related and EEG-related models. In the new method (Fig. 12), another adversarial sample ${E}^{adv}$, which is aggressive to EEG-related models and could be generated with any existing method, is used to modify the perturbation of rhythm power array ${\eta }_{P}$ and then to help the generation of final EEG adversarial sample *É* by replacing information from the raw ${\varvec{E}}$.

In the experiment, FGSM is used to generate ${E}^{adv}$ from EEG-related models and GPBEAM/GPBEAM-DE(*N* = 5) is used to generate perturbation of rhythm power array ${\eta }_{P}$ from BEAM-related models. Here, ${\epsilon }_{E}$ denotes the parameter $\epsilon$ of FGSM when it is used for ${E}^{adv}$ and ${\epsilon }_{B}$ denotes the parameter $\epsilon$ of FGSM when it is used for ${\eta }_{P}$ in GPBEAM/GPBEAM-DE.

To keep the adversarial samples imperceptible and to make it easy to compare the aggressive performance of the methods in this paper before and after the modification, this experiment keep the DL of the adversarial samples produced by the methods in this paper before and after the modification unchanged. That is, this experiment first use GPBEAM/GPBEAM-DE to generate adversarial samples and get their DL value, then run modified GPBEAM/GPBEAM-DE to generate new adversarial samples that have the same DL value by adjusting the ${\epsilon }_{E}$ parameter.

Table 8 shows the performance of the GPBEAM/GPBEAM-DE (without the addition of ${\mathrm{E}}^{\mathrm{adv}}$). Table 9 shows the performance of the modified GPBEAM/GPBEAM-DE (with the addition of ${E}^{adv}$). It is clear that by the modification, GPBEAM/GPBEAM-DE obtained the new ability of attacking EEG-related models, with the top attack success rate changed from 0.03 to 0.64 and the minimum attack success rate changed from 0.01 to 0.11. The modification does not change the power of GPBEAM/GPBEAM-DE for attacking BEAM-related model. It should be noted that the capacity enhancement of the modified GPBEAM/GPBEAM-DE mainly attribute to the adding of the adversarial sample ${E}^{adv}$, and this paper just propose a way to fuse the information of the added adversarial sample for attacking EEG-related models and information of the adversarial sample for attacking BEAM-related models in the framework of GPBEAM/GPBEAM-DE. Furthermore, the improvement would have been better if a more aggressive perturbation generation algorithm had been used to generate ${E}^{adv}$.

**Table 9:** Performance of the modified methods in the case of maintaining the same ${DL}_{E}$ as Table 5. The parameter N for the modified GPBEAM-DE is set to be 5

Architecture				Maxpool		Temporal convolution		LSTM		Mixed LSTM	
${\mathrm{DL}}_{E}$	${\epsilon }_{B}$	${\epsilon }_{E}$	Evaluation Criteria	Acc	SR	Acc	SR	Acc	SR	Acc	SR
EEG-related models											
0	-	-	-	0.92	-	0.84	-	0.90	-	0.88	-
0.024	0.1	0.023	modified GPBEAM	0.42	0.49	0.28	**0.55**	0.71	0.18	0.75	0.14
0.021	0.1	0.020	modified GPBEAM-DE	0.47	0.41	0.34	**0.50**	0.77	0.15	0.77	0.11
0.030	0.3	0.029	modified GPBEAM	0.36	0.55	0.23	**0.61**	0.68	0.21	0.73	0.16
0.026	0.3	0.025	modified GPBEAM-DE	0.40	0.51	0.26	**0.58**	0.70	0.20	0.75	0.14
0.036	0.5	0.035	modified GPBEAM	0.34	0.58	0.19	**0.64**	0.67	0.23	0.69	0.19
0.030	0.5	0.029	modified GPBEAM-DE	0.36	0.55	0.23	**0.61**	0.68	0.21	0.71	0.17
BEAM-related models											
0	-	-	-	0.92	-	0.92	-	0.93	-	0.94	-
0.024	0.1	0.023	modified GPBEAM	0.43	0.50	0.37	**0.55**	0.76	0.18	0.73	0.20
0.021	0.1	0.020	modified GPBEAM-DE	0.61	0.29	0.51	**0.41**	0.58	0.37	0.64	0.31
0.030	0.3	0.029	modified GPBEAM	0.18	0.75	0.17	**0.75**	0.61	0.33	0.61	0.32
0.026	0.3	0.025	modified GPBEAM-DE	0.28	0.67	0.25	**0.71**	0.26	0.70	0.25	0.70
0.036	0.5	0.035	modified GPBEAM	0.12	0.75	0.12	**0.80**	0.58	0.36	0.56	0.37
0.030	0.5	0.029	modified GPBEAM-DE	0.14	0.78	0.13	**0.80**	0.17	0.79	0.15	0.80

## Conclusion

This paper examines the vulnerability of deep learning models for diagnosing epilepsy to white-box attacks. It proposes two methods, GPBEAM and GPBEAM-DE, which generate EEG adversarial samples by perturbing BEAMs densely and sparsely respectively. Unlike existing studies that generate EEG adversarial samples by perturbing raw EEG signal、EEG frequency and EEG spectrograms, this paper generates EEG adversarial samples by perturbing BEAMs for the first time. This study exposes an important safety issue for brain disease diagnostic systems with experiments using EEG data from the CHB-MIT dataset and two types of victim models each of which has four different DNN architectures.

The experimental results show that: (1) GPBEAM/GPBEAM-DE can successfully attack all BEAM-related models with either pure CNN architectures or CNN + RNN architectures, showing their strong aggressiveness; (2) The aggressiveness of GPBEAM is sensitive to the effectiveness of the perturbation generation part which can theoretically be any white-box attack. It shows another merit of GPBEAM that its performance could be further improved by introducing new state-of-arts perturbation generation method other than any of those methods (FGSM, I-FGSM, MI-FGSM, DII-FGSM, PGD and C&W) having tested in this paper; (3) The sparse attack method GPBEAM-DE outperforms the dense attack method GPBEAM in both SR and DL in most cases. That is because of the novel work, the combination of GPBEAM, DE and perturbation overflow in GPBEAM-DE. DE is used to directly perturb some elements of the rhythmic power array. With the help of the sign information of the perturbation generated by GPBEAM, when the magnitude of the perturbation generated by DE exceeds a predefined level, the excess is allocated to other electrodes by perturbation overflow; (4) By using perturbation overflow, at the expense of a certain degree of distortion, the attack power of GPBEAM-DE can be increased significantly; (5) Among four BEAM-related models with different neural network architecture, the adversarial samples generated by GPBEAM/GPBEAM-DE have obvious transferability.

There are some limitations that must be considered, before using the proposed methods to accomplish attacking tasks. Currently, the proposed methods could only work in the digital-domain. They could have the chance to perturb EEG data and deceive models only if (1) there are time lags between the finish of capturing EEG data and that the victim deep-learning models start processing those data, (2) these EEG data could be stolen by hacking, and (3) these victim models are white-boxes to attackers (means that attackers have copies of these models and could use them to calculate perturbations). Using them in physical-domain will face some other limitations as mentioned by Dongrui Wu et al. [11]. They are (1) Trial-specificity, i.e., the attacker needs to generate different adversarial perturbations for different EEG trials; (2) Channel-specificity, i.e., the attacker needs to generate different adversarial perturbations for different EEG channels; (3) Non-causality, i.e., the complete EEG trial needs to be known in advance to compute the corresponding adversarial perturbation; (4) Synchronization, i.e., the exact starting time of the EEG trial needs to be known for the best attack performance.

Although GPBEAM-DE obtains better performance than GPBEAM in most attacking cases, it has some limitations should be noted. First of all, GPBEAM-DE needs feedback on whether the attack is successful during the execution of the evolutionary algorithm, which requires getting the labeled EEG data in advance. Secondly, the evolutionary algorithm itself requires much time to converge. Furthermore, unlike GPBEAM, which could easily create universal adversarial perturbations by using a universal perturbation generation algorithm as its part, GPBEAM-DE could not create universal adversarial perturbations easily. At last, GPBEAM-DE is a bit worse than GPBEAM in transferability. The adversarial samples generated by the methods in this paper show almost no aggressiveness to the four EEG-related models in the experiments, indicating a poor transferability from BEAM-related models to EEG-related models; At last, a simple modification to the GPBEAM/GPBEAM-DE will make it have aggressiveness to both BEAM-related and EEG-related models, and this capacity enhancement is done without any cost of distortion increment.

There are many further works which could be done in the future, such as: (1) The perturbation generation algorithms used for GPBEAM/GPBEAM-DE could theoretically be replaced by any of other state-of-art ones for pursuing better performance or new features; (2) Instead of white-box scenario, the black-box scenario, which is of greater significance to the security of BCI in real world, should be considered; (3) Although the proposed attacks do not be affected by EEG-to-BEAM transformation, whether they still be effective after commonly-used EEG preprocessing which is an important part of BCI pipeline, is worth studying; (4) More aggressive and imperceptible attacks could be produced by making them sparse in all time, rhythm, and electrode dimensions.

It should be claimed that the goal of this study is not to attack any of the EEG medical diagnostic systems, but to raise concerns about the safety of deep learning models and hope to lead us to a safer design.

## Data Availability

*Source of data:* Public access to the database(s) is open. A team of investigators from Children’s Hospital Boston (CHB) and the Massachusetts Institute of Technology (MIT) created and contributed this database to PhysioNet. https://archive.physionet.org/physiobank/database/chbmit/ This database is described in Ali Shoeb. Application of Machine Learning to Epileptic Seizure Onset Detection and Treatment. PhD Thesis, Massachusetts Institute of Technology, September 2009. DOI for CHB-MIT Scalp EEG Database: 10.13026/C2K01R *The code can be referred to:* https://github.com/yyyuuu060/perturbing-BEAMs.git
